# Hematopoiesis and innate immunity: an inseparable couple for good and bad times, bound together by an hormetic relationship

**DOI:** 10.1038/s41375-021-01482-0

**Published:** 2021-12-01

**Authors:** Mariusz Z. Ratajczak, Magdalena Kucia

**Affiliations:** 1 Stem Cell Institute at Graham Brown Cancer Center, University of Louisville, Louisville, KY USA; 2 Laboratory of Regenerative Medicine Medical University of Warsaw, Warsaw, Poland

**Keywords:** Stem cells, Immunology

## Abstract

Hematopoietic and immune cells originate from a common hematopoietic/lymphopoietic stem cell what explains that these different cell types often share the same receptors and respond to similar factors. Moreover, the common goal of both lineages is to ensure tissue homeostasis under steady-state conditions, fight invading pathogens, and promote tissue repair. We will highlight accumulating evidence that innate and adaptive immunity modulate several aspects of hematopoiesis within the hormetic zone in which the biological response to low exposure to potential stressors generally is favorable and benefits hematopoietic stem/progenitor cells (HSPCs). Innate immunity impact on hematopoiesis is pleiotropic and involves both the cellular arm, comprised of innate immunity cells, and the soluble arm, whose major component is the complement cascade (ComC). In addition, several mediators released by innate immunity cells, including inflammatory cytokines and small antimicrobial cationic peptides, affect hematopoiesis. There are intriguing observations that HSPCs and immune cells share several cell-surface pattern-recognition receptors (PRRs), such as Toll-like receptors (TLRs) and cytosol-expressed NOD, NOD-like, and RIG-I-like receptors and thus can be considered “pathogen sensors”. In addition, not only lymphocytes but also HSPCs express functional intracellular complement proteins, defined as complosome which poses challenging questions for further investigation of the intracellular ComC-mediated intracrine regulation of hematopoiesis.

## Introduction

It is well known that hematopoietic and immune cells originate from a common hematopoietic/lymphopoietic stem cell [1, 2]. However, these cells are studied by different interest groups, hematologists and immunologists, respectively, who in most cases focus on the biological functions of only one of these lineages. Nevertheless, in this perspective review, we will present findings that show that hematopoietic and immune cells have intensive crosstalk during development and under steady-state conditions and, in particular, cooperate in response to stress/danger challenges. We will focus mainly on innate immunity, which is the oldest part of the immune system, which, by engaging adaptive immunity cells may directly or indirectly influence hematopoiesis [3, 4].

Deciphering these interactions will lead to a better understanding of bone marrow (BM) responses to tissue and organ damage, infections, and the onset of emergency hematopoiesis [5]. In these situations, danger-associated molecular pattern (DAMP) and pathogen-associated molecular pattern (PAMP) molecules are released, which are recognized by pattern-recognition receptors (PPRs) expressed not only by immune cells but also by hematopoietic stem/progenitor cells (HSPCs) [3, 6–8]. Activation of PRRs and triggering of the complement cascade (ComC) leads to fine-tuned immune responses and, in some cases, emergency myelopoiesis [9]. During chronic infections, these processes might impinge on HSPCs and lead to aging of these cells [7, 10] and the development of hematopoietic disorders [11].

We will also discuss the emerging role of innate immunity as a central orchestrator regulating the circulation of HSPCs in response to the intrinsic circadian rhythm clock [12] and the trafficking of these cells during pharmacological mobilization and hematopoietic transplantation [13, 14]. Therefore, these results are relevant for better understanding and optimizing clinical protocols for hematopoietic transplantations and will, on the one hand, allow better mobilization of these cells from BM into peripheral blood (PB) as a source of HSPCs for a hematopoietic graft and, on the other hand, improve their seeding efficiency into recipient BM after transplantation. Importantly, innate immunity, in addition to HSPCs, also affects different types of BM-residing non-hematopoietic stem cells.

Finally, recent breakthrough research revealed that not only PPRs but also certain ComC proteins and receptors are expressed intracellularly in T lymphocytes, regulate several aspects of their metabolism, and play an important role in maintaining the functional robustness of these cells. The presence of functional autocrine components of the ComC inside T cells has been described as the “complosome” [15–18]. This finding changed our view of ComC-mediated regulation of immune responses. Interestingly, recent research from our group indicates that the complosome is also operational in normal HSPCs [8], which suggests the involvement of even more significant mutual interactions between hematopoiesis and innate immunity than envisioned so far.

### Cellular and soluble arms of innate immunity

The innate immune system and the adaptive immune system play pivotal roles in the defense against pathogens in vertebrates. However, from a developmental point of view, the innate immune system is evolutionarily older. The overall function of innate immunity is to *i)* recruit all types of immune cells to sites of infection, *ii)* activate the ComC to identify and bind invading pathogens and subsequently engage effector cells, *iii)* remove foreign substances present in the tissues, and *iv)* promote responses by the adaptive immune system [19–21].

While the cellular arm of innate immunity consists of granulocytes, macrophages, dendritic cells, NK cells, γδ-T cells, and recently identified innate lymphoid cells (ILCs), the most important components of the soluble arm are the complement cascade (ComC) proteins [20]. While the role of classical innate immunity cells (e.g., granulocytes or macrophages) has been studied extensively, ILCs still need further investigation. Some researchers consider these cells to be innate counterparts of T cells, which originate from a common lymphoid progenitor and are characterized by an absence of regular lymphoid morphology, an absence of the *RAG* gene (required for receptor rearrangement in B and T cells), and expression of markers present on the surface of myeloid and dendritic cells [22]. The ComC, part of the soluble arm of innate immunity, is activated by the classical, mannan-binding lectin, and the alternative pathways [19–21]. Activation of the ComC is triggered by certain DAMPs and PAMPs, as will be discussed below [20, 23].

While under steady-state conditions, innate immunity orchestrates a circadian circulation of HSPCs in PB [24–27], activation of innate immunity components (as seen during the administration of pharmacological drugs) promotes forced egress or mobilization of HSPCs from BM into PB [13, 14] and facilitates HSPC homing and engraftment into the recipient BM after myeloablative conditioning for transplantation by chemo- or radiotherapy [28–32]. We proposed that both pharmacological mobilization and myeloablative conditioning for transplantation induce an innate immunity-mediated state of “sterile inflammation” in BM that directs effective egress of HSPCs from BM and their migration to BM stem cell niches after transplantation [33, 34]. Thus, we will focus in this perspective review on the critical role of innate immunity as an orchestrator of HSPC trafficking. We will also discuss the role of intracellular complement (the complosome), which was initially identified in lymphocytes [15–18] and recent evidence shows is also present in HSPCs [8]. This evidence sheds new light on intracrine complement involvement in regulating the metabolism and proliferation of HSPCs.

What is important for this perspective review, many beneficial homeostatic effects of innate immunity on HSPCs or the hematopoietic BM microenvironment can be defined as biological effects within the “hormetic zone” [8, 35, 36]. Hormesis is a characteristic of many biological processes when there is exposure to increasing amounts of challenging or potentially harmful stimuli. The effects are biphasic: while a low dose of a challenging agent can be beneficial to cells, a high dose can be damaging. Interestingly, there is generally a favorable biological response to low exposures to a potential stressor—in what is called the “hormetic zone”—and several beneficial effects of innate immunity on hematopoiesis can be explained by this phenomenon [8, 35, 36]. We will return to this concept while discussing the biological effects of ComC and Nlrp3 inflammasome activation, which seem to be favorable for HSPCs within the hormetic zone [8].

**Fig. 1:**
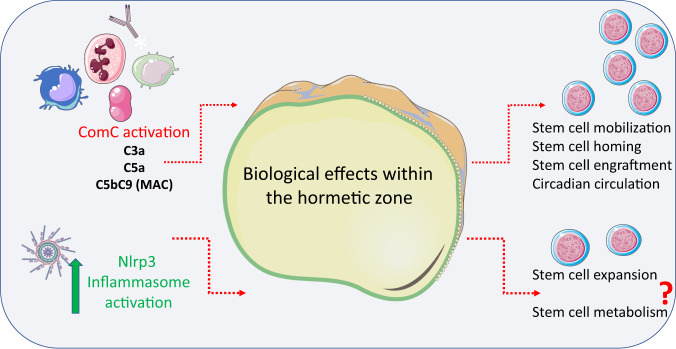
Effects of innate immunity when activated within the hormetic zone on the surface of HSPCs. Mediators of innate immunity, including the ComC-derived anaphylatoxins C3a, C5a, and C5b-C9 at low concentration and activation of Nlrp3 inflammasomes within the hormetic zone, exert several positive effects on hematopoiesis, including (i) regulation of HSPC circadian circulation, (ii) mobilization in response to external and intrinsic stimuli, (iii) homing and engraftment after transplantation, and, what is also very likely, (iv) promotion of HSPC proliferation and expansion due to regulation of intracellular metabolism in a complosome-dependent manner.

It is also important to keep in mind that multiple checkpoints regulate activation of innate immunity to maintain the beneficial effects of its mediators acting within the hormetic zone and to prevent damaging effects. For example, there are receptors on the cell surface that ameliorate the potential harmful effects of soluble activated ComC cleavage products (e.g., C3a and C5a) and solid-phase mediators (e.g., iC3b). Interestingly, evidence has accumulated that the final ComC activation product, C5b-C9 (also known as soluble membrane attack complex, sMAC), is endowed with cell lytic activity. In some cases, instead of damaging cells, this complex stimulates them [20, 37]. The general idea of hormetic effects of innate immunity elements on HSPCs is depicted in Fig. 1.

Moreover, in order to keep a balance, the activation of innate immunity may be damped by anti-inflammatory effects of heme oxygenase 1 (HO-1) [38–41]. In addition, an important role is played by the cell surface glycoprotein CD59, also known as MAC-inhibitory protein (MAC-IP, also known as protectin), which prevents the formation of fully active lytic MAC [19, 42], and CD55 (also known as complement decay-accelerating factor, DAF), which regulates the complement system on the cell surface by preventing the formation of C3 convertase during ComC activation and MAC formation [19, 42]. Another receptor, CD46 (also known as complement regulatory protein or membrane cofactor protein), which is expressed by human cells and is also an inhibitory complement receptor [43]. Interestingly, this receptor, which will be discussed further, has another function in complosome-mediated effects on hematopoiesis [15–18]. It is important to remember that all these receptors are expressed not only on the surfaces of innate immune cells but also on HSPCs to protect them from hyperactivated ComC products acting outside the hormetic zone. Finally, HSPCs also express other surface inhibitory molecules to evade innate and adaptive immunity attacks, including CD47, that serve as a “*don’t eat me*” signal to the immune system’s macrophages [44].

### The emerging role of innate immunity mediators in regulating hematopoiesis

From an historical point of view, the classical hematopoietic regulators comprise peptide-based cytokines, chemokines, and growth factors [45]. Nevertheless, over time, other non-peptide regulators have been identified, including extracellular signaling nucleotides, such as extracellular adenosine triphosphate (eATP), the most essential member of this family [46], and two potent phosphosphingolipids, sphingosine-1-phosphate (S1P) and ceramide-1-phosphate (C1P) [47–49]. Besides the α-chemokine stromal-derived factor 1 (SDF-1), these mediators regulate the migration of HSPCs. Interestingly, ComC cleavage fragments, such as C3a or C5a, do not directly chemoattract HSPCs [45]. C3a and certain other mediators released from activated innate immune cells (including cationic antimicrobial peptides such as LL-37, an active fragment of cathelicidin, and β2-defensin) enhance the chemotactic responsiveness of HSPCs to SDF-1, S1P, and eATP gradients [50, 51]. As demonstrated in the case of SDF-1, this effect depends on the incorporation of its receptor, CXCR4, into membrane lipid rafts (MLRs) [28, 33], which are domains of the plasma membrane enriched for glycosphingolipids, cholesterol, and certain protein receptors. These specialized membrane microdomains serve as membrane organizing centers, allowing a closer interaction of protein receptors and their corresponding ligands to promote optimal signal transduction. Thus, C3a, LL-37, and β2 defensin, which are mediators of innate immunity, have been identified as important modulators that increase MLR formation and thus promote the chemotactic responsiveness of HSPCs to SDF-1 gradient [50–54].

Although HSPCs express receptors for C3a and C5a anaphylatoxins (C3aR, and C5aR1 and C5aR2, respectively) on their surface, they do not respond by chemotaxis to these ComC cleavage fragments [45]. In contrast to HSPCs, granulocytes and macrophages migrate robustly in response to C3a and C5a gradients [55]. This behavior plays an important role in pharmacological- or stress-induced egress of HSPCs from BM into PB [26, 56]. As demonstrated, during the mobilization process, granulocytes are chemoattracted first into PB in response to C5a released during ComC activation in the circulation. An elevated C5a level in PB chemoattracts granulocytes, which release proteolytic enzymes and are first to cross the BM–PB endothelial barrier. This crossing facilitates subsequent egress of HSPCs, which are chemoattracted by a steep S1P gradient in PB and follow in the footsteps of the granulocytes [26, 56]. In this way, regulation of the egress/mobilization of HSPCs is tightly coordinated by cellular (granulocytes) and the soluble arm (C5a) of innate immunity.

In addition, to ComC cleavage fragments and small antimicrobial cationic peptides, several mediators released by innate immunity cells, including TNF-α; INF-α, β, and γ; IL-1β; and IL-6, affect hematopoiesis. The principal sources of these factors are macrophages, neutrophils, NK cells, and BM stromal cells. Both inhibitory and stimulatory pleiotropic effects of these cytokines on the proliferation and survival of HSPCs have been described. For example, TNF-α shows a differential impact on HSPCs, depending on whether they are quiescent or cycling [57]. In addition, as recently demonstrated, while TNF-α induces myeloid progenitor apoptosis, at the same time it promotes HSPC survival and myeloid differentiation [57]. Interferons type I (INF-α, β) have been demonstrated to positively affect HSPCs, facilitating their transition from quiescence to proliferation [58].

By contrast, interferon γ negatively affects HSPC homeostasis, forcing their differentiation over self-renewal [59]. The hematopoietic effects of IL-1β, which is released from cells in an Nlrp3 inflammasome-dependent manner, are visible at the level of myelopoiesis, as this cytokine increases the production of promyelocytic cytokines by BM accessory cells [60]. On the other hand, IL-6 decreases secretion of erythropoietin in the kidney [60, 61] and thus negatively affects erythropoiesis. Overall, proinflammatory cytokines are involved in the pathogenesis of chronic disease anemia [62]. This is an example of how prolonged innate immunity-mediated inflammation becomes harmful to hematopoiesis.

### Pattern-recognition receptors and hematopoiesis

Pattern-recognition receptors (PRRs) are host innate immunity sensors that detect pathogen-derived PAMPs and other molecules, known as DAMPs or alarmines, that are associated with the host’s own damaged or dying cells [6]. Thus, PPRs serve to eliminate pathogenic intruders and are an integral part of tissue homeostasis, involving sterile non-inflammatory removal of damaged cells and in this way facilitating tissue repair. PRRs can be divided into receptors expressed on the cell surface, including Toll-like receptors (TLRs) and C-type lectin receptors [3, 6, 63], and those expressed in the cytoplasm, including NOD-like receptors (NLRs) and RIG-I-like receptors [6, 24]. ComC proteins, along with collectins and ficolins, circulating in PB form the soluble arm of innate immunity and can be considered as soluble circulating PRRs. The most crucial, collectin, is a mannan-binding lectin (MBL) [64]. Among PRRs, evidence has accumulated that TLRs and NLRs, including NOD1, NOD2, and NOD-like receptors, such as Nlrp1-, Nlrp3-, Nlrp12-, and AIM2-inflammasomes, have an impact on hematopoiesis. While TLRs are activated by several PAMPs and DAMPs [3, 6, 63], NOD1 and NOD2 receptors are activated by PAMPs, which are bacterial peptidoglycans [65]. By contrast, the intracellular Nlrp3 inflammasome is activated mainly through DAMPs [66–68]. The most potent stimulators of Nlrp3 inflammasomes are *i)* eATP (as mentioned above), *ii)* reactive oxygen species (ROS), and *iii)* ComC cleavage products (C3a, C5a, and C5b-C9) [66–68]. Activation of Nlrp3 inflammasomes in response to eATP signaling established these PRRs as purinergic signaling effectors. By contrast, the AIM2 inflammasome is activated by irradiation-damaged DNA [69]. We will highlight what is already known about the role of PRRs in hematopoiesis below.

*-Toll-like receptors (TLRs)*. These PRRs activate immune responses and are primarily highly expressed on the surfaces of macrophages and dendritic cells [3, 6, 63]. They are also present on the surfaces of cells in the hematopoietic microenvironment, including stromal cells and endothelial cells. Several members of this receptor family are also found on HSPCs [3, 6, 63]. Overall, there are 9 TLR members in humans and 13 in mice, and most are predominantly expressed on cell-surface membranes (TLR1-6 and TLR10); however, some are located within intracellular endosomes (TLR3 and TLR7-9). The most relevant receptor for the topic of this spotlight is TLR4, which is activated by Gram-negative bacteria-derived liposaccharide (LPS), and TLR9, which is activated by nucleic acids. TLRs play an important role in regulating the differentiation of HSPCs into innate immunity cells of both myeloid and lymphoid origin. TLRs orchestrate emergency hematopoiesis to supply the proper repertoire of effector cells and provide pro-inflammatory cytokines [63]. However, prolonged stimulation of TLRs reportedly impairs the engrafting ability of HSPCs, and, as discussed below, contributes to HSPC aging and increases the chance of malignant transformation [70, 71]. Except for TLR3, most TLRs utilize the MyD88 adapter protein to activate the transcription factor NF-κb, which is responsible for the expression of various pro-inflammatory cytokines (TNF-α; INF-α, β, and γ; IL-1β; and IL-6) as well as chemokines and adhesion molecules [3, 6], which together trigger acute inflammation and stimulation of adaptive immunity. For expression of type I interferons (INF-α, β), TLR4 also utilizes a TIR domain-containing, adapter-inducing transcription factor, interferon regulatory factor 3 (IRF3) [63]. Since LPS is released from Gram-negative intestinal bacteria and circulates in PB after absorption from the intestine, TLR4 is continuously activated [63]. As mentioned above, TLRs play an important role during “emergency hematopoiesis,” providing leukocytes and macrophages to fight infection. These effects are mediated by TLRs expressed on cells in the hematopoietic microenvironment. Still, experiments performed in TLR2^–/–^, TLR4^–/–^, or MyoD88^–/–^ mice reconstituted with wild type (WT) HSPCs and stimulated with their respective TLR agonists revealed that, despite a lack of functional TLRs in the BM stroma of chimeric mice, WT HSPCs responded to TLR2 agonists and differentiated preferentially into macrophages [72]. Thus, TLRs expressed on HSPCs instruct these cells to specify into cells fighting infection. As will also be discussed below, LPS circulating in PB after release from Gram-negative intestinal bacteria maintain, by engaging TLR4, the expression of Nlrp3 inflammasomes in innate immunity cells and in HSPCs. This interaction leads to the transcription of Nlrp3 inflammasome components in an NF-κb-dependent manner and maintains their basic level of expression [66–68]. This interplay between intestine-derived LPS and baseline expression of the Nlrp3 inflammasome has become relevant in light of recent studies on the role of the intestinal microbiome (microbiota) in body homeostasis. Interestingly, recent results indicate that stimulation with specific ligands of TLR7 and TLR8 on the surface of human CD34^+^ cells (enriched in HSPCs) increases proinflammatory cytokine production and forces these cells to differentiate into macrophages and dendritic cells, which further supports the idea that HSPCs can themselves serve as pathogen sensors [3, 72]. Nevertheless, further studies are needed to dissect which effects of TLR stimulation on hematopoiesis depend on autocrine and which on paracrine signaling.
- *NOD1 and NOD2 receptors*. The nuclear-binding oligomerization domain 1 and 2 (NOD1 and NOD2) PRRs are activated by PAMPs in response to bacterial peptidoglycans [6]. As reported, these receptors, in synergy with TLR4, mobilize HSPCs during infection, promoting their egress from BM into PB and subsequently their homing into the spleen, which plays an important role as an extramedullary place of hematopoiesis during stress situations [6]. To better address this issue, in mobilization studies the authors employed TLR4^–/–^ mice and mice lacking expression of adapter protein receptor-interacting serine–threonine kinase 2 (RIPK2^–/–^), which is involved in NOD1 and NOD2 signaling [65]. Activation of these receptors decreased the level of SDF-1 involved in the retention of HSPCs in BM microenvironment stem cell niches and led to upregulation of endogenous granulocyte colony stimulating factor (G-CSF). This cytokine was required for mobilization of HSPCs from BM and their extramedullary accumulation in the spleen. HSPCs mobilized in response to NOD1/NOD2 and TLR4 activation in the spleen gave rise to neutrophils and monocytes [65]. These effects, however, depended mainly on the activation of NOD1 and NOD2 receptors in nonhematopoietic cells in the BM microenvironment. Moreover, NOD1 and NOD2 signaling acted synergistically with TLR4, and expression of TLR4 on the surface of HSPCs was not required for splenic accumulation of these cells [65].
- *NOD-like or NLRP (nucleotide-binding oligomerization domain, leucine rich repeat and pyrin domain-containing)* receptors. There are at least 14 members of this family of receptors that play a role in immune responses. These PRR members are activated by canonical or non-canonical pathways and were initially identified in immune cells, as some of them trigger immune responses [66–68]. In this spotlight review we will focus on the Nlrp3, Nlrp1, Nlrp12, and AIM2 inflammasomes, as they have been reported to play a role in hematopoiesis.
- *Nlrp3 inflammasome*. This NOD-like receptor is, as mentioned above, also expressed and functional in HSPCs [7, 8]. It is activated by DAMPs, including extracellular adenosine triphosphate (eATP), which links this particular PRR with purinergic signaling and explains at the molecular level the role of eATP as an important regulator of hematopoiesis [46]. Furthermore, the Nlrp3 inflammasome is strongly activated by ComC cleavage fragments (C3a and C5a), not only in innate immunity cells but also in HSPCs [23, 66–68, 73]. In addition, the Nlrp3 inflammasome may also be activated by the C5b-9 sublytic membrane attack complex (MAC) [74]. The ComC itself is considered by some investigators to be a circulating liquid-phase PRR and, besides pathogen-derived antigens, the ComC is activated in response to several DAMPs, including eATP and high mobility group box 1 (HMGB-1) protein [75]. The Nlrp3 inflammasome is also activated by reactive oxygen species (ROS) released from cell membrane-associated NADPH oxidase 2 (Nox2) [66–68, 76]. Nox2 releases more ROS when cell membrane-expressed receptors involved in migration of HSPCs are engaged by specific ligands, as demonstrated for CXCR4 receptor activation by SDF-1, the S1P receptor by S1P, and the P2X4 and P2X7 receptors by eATP. This pattern of activation provides further evidence for crosstalk between hematopoietic regulators and the Nlrp3 inflammasome. Because of this crosstalk, the Nlrp3 inflammasome promotes trafficking of HSPCs, as seen during mobilization and homing/engraftment after transplantation [28, 76, 77]. The Nlrp3 inflammasome is also released from cells in the form of particles that, after internalization by surrounding cells, promote the spread of the immune response [78]. Finally, the Nlrp3 inflammasome is also strongly activated by mitochondria-derived ROS. We mentioned above the role of intestinal bacteria-derived LPS in priming the basic expression level of Nlrp3 inflammasomes in the cells in a TLR4–sNF-κB-dependent manner. However, hyperactivation of Nlrp3 inflammasomes outside the hormetic zone may lead to cell death by the mechanism of pyroptosis [66–68], a caspase-1-mediated process that leads to the formation of gasdermin pores in the cell membrane and leakage of the cytosol contents into the extracellular space. Nlrp3 inflammasome activation releases IL-1β, which is also an important stimulator for the expression of several proinflammatory cytokines, including TNF, IL-6, and IL-1α. This stimulation explains why activation of Nlrp3 inflammasomes in response to COVID19 infection leads to a cytokine storm and damage to HSPCs [79–81]. Nevertheless, the intriguing question remains whether activation of Nlrp3 inflammasomes within the hormetic zone promotes steady-state proliferation and post-transplantation expansion of HSPCs. This question will be discussed later in this spotlight.
- *Nlrp1 inflammasome*. Overall activation of this PRR leads to hematopoietic cell death by pyroptosis, and thus is detrimental for hematopoiesis [82]. Three paralogs of the *Nlrp1* gene (*Nlrp1a, b, c*) have been identified. While, *Nlrp1c* is a pseudogene, the two other genes are functional. Nlrp1b is activated in mice by proteolytic cleavage, but the mechanism of activation for Nlrp1a is unclear at the moment. In a recently published report, it was demonstrated, using *Nlrp1b*-KO mice, that expression of this gene in bone marrow endothelial and stromal cells inhibits the engraftment of HSPCs and hematopoietic reconstitution after transplantation [83]. In support of this finding, the recovery of BM and PB counts was enhanced in *Nlrp1*-KO animals. Thus, Nlrp1b in the bone marrow microenvironment inhibits the engraftment of hematopoietic stem progenitor cells and hematopoietic reconstitution after transplantation. However, further studies are needed to see whether Nlrp1b is also expressed in HSPCs and how its deficiency affects the navigation of *Nlrp1b*-KO cells to BM. In parallel, it is important to perform mobilization studies in these mice and to assess the effect of another paralog, *Nlrp1a*, in HSPC mobilization and homing. It is known that various members of the NOD-like family of receptors have different biological effects, and some promote, while others inhibit, inflammation [84, 85]. These inhibitory NOD-like receptors, for example, NLRP12, NLRC3, and NLRX1, attenuate diverse signaling pathways involving NF-κB and type I interferon (IFN) signaling, together with cellular processes such as generation of reactive oxygen species (ROS) and autophagy [83–85].
-*Nlrp12 inflammas*ome. This PRR has some beneficial effects on HSPCs [7] and may inhibit the negative effects of TNF-α during emergency hematopoiesis. Specifically, overexpression of Nlrp12 accelerates myelopoiesis and immune reconstitution in BM exposed to stress stimuli [7].
- *AIM2 (absent in melanoma 2) inflammasome*. This damaged double-stranded, DNA-sensing PRR controls radiation-induced cell death and tissue injury [69]. *AIM2*-KO mice are somewhat protected from irradiation-induced hematopoietic failure.

Based on these findings, PRRs have a pleiotropic effect on hematopoiesis, either beneficial or detrimental, and more work is needed to decipher their role in normal and pathological hematopoiesis. In particular, it is essential to assess the biological effects of other NOD-like receptors in addition to Nlrp1, Nlrp3, Nlrp12, and Aim2. We can assume that there exists some redundancy in their biological responses.

### HSPCs “patrol” peripheral tissues and locally supply cells to fight infection

For many years it has been proposed that innate immune cells, such as granulocytes or monocytes, originate in BM and are released into PB to fight infections. Under steady-state conditions, they circulate in PB during their short lifetime, ready to engage potential microbial invaders. However, recent evidence demonstrated that, in addition to granulocytes, HSPCs committed to the granulocyte–monocytic lineage are released under steady-state conditions into the lymphatics and PB to patrol peripheral tissues for potential intruders [86]. It has been shown that such circulating progenitors proliferate and supply granulocytes and monocytes directly to the affected tissues as needed. This response during systemic or local infection is mediated mainly by interleukin 1 beta (IL-1β), a known pyrogen released in response to activation of TLR4 and the Nlrp3 inflammasome. While migration of these cells toward potential “battlefields” is regulated by locally released HSPC chemoattractants, granulocytes and monocytes accumulate in infected tissues in response to ComC cleavage fragments [86, 87]. Thus, this important self-defense mechanism requires a well-coordinated response from innate immunity cells, PRRs, and circulating HSPCs.

### Mobilization and homing/engraftment of hematopoietic stem cells are regulated by innate immunity cells

Innate immunity plays an important role in the trafficking of HSPCs, as seen during pharmacological mobilization of these cells and their navigation to BM after hematopoietic transplantation, followed by homing and engraftment [88–90].

**Fig. 2:**
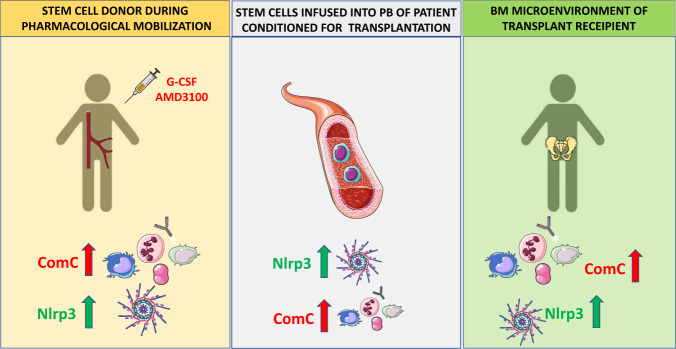
Innate immunity, including activation of the ComC and the Nlrp3 inflammasome, orchestrates different steps of hematopoietic transplantation. Innate immunity mediators, including ComC-derived anaphylatoxins C3a, C5a, and C5b-C9 and the Nlrp3 inflammasome, are important for inducing sterile inflammation in the donor BM during pharmacological mobilization of HSPCs and in the recipient BM after myeloablative conditioning for transplantation by radio/chemotherapy. As depicted here, ComC cleavage fragments and the Nlrp3 inflammasome promote optimal egress of HSPCs from BM into PB (**A**), promote navigation of HSPCs to BM niches (**B**), and activate the ComC and expression of Nlrp3 inflammasomes in the BM microenvironment of a transplantation recipient (**C**), all of which are required for proper homing and engraftment of transplanted cells.

*-Mobilization of HSPCs*. HSPCs reside in stem cell niches in the BM microenvironment and stay quiescent. Nevertheless, they divide at a low rate, and because of limited stem cell occupancy, some of them have to leave the niche, enter the PB, and circulate to find new niches to occupy [45]. This circulation follows certain circadian rhythm changes regulated by innate immunity that will be discussed in this spotlight [27]. The number of HSPCs circulating in PB increases during inflammation and tissue or organ injuries and could increase up to 100 fold after administration of pro-mobilizing drugs, such as granulocyte colony-stimulating factor (G-CSF) or an antagonist of the CXCR4 receptor, AMD3100 (also known as plerixafor) [13, 14]. During mobilization, HSPCs are released from their BM niches and migrate across the BM–PB endothelial barrier into BM sinusoids. Several redundant mechanisms regulate this process, but, as we have proposed, activation of the ComC through the mannan-binding lectin (MBL) and alternative activation pathways plays a critical role [30, 82]. Mice that are C5 deficient (C5^–/–^) or that do not activate the MBL and alternative ComC activation pathways (MBL^–/–^ and FB^–/–^, respectively) are poor mobilizers of HSPCs [30, 82]. Our results demonstrated that the administration of pro-mobilizing drugs leads to activation of the ComC by two different mechanisms. First, pro-mobilizing agents induce sterile inflammation in the BM microenvironment, which exposes a neoepitope that is recognized by naturally occurring IgM antibodies, thereby activating the ComC [30]. At the same time, pro-mobilizing agents release eATP from BM cells, which, via the P2X7 and P2X4 purinergic receptors, activates Nlrp3 inflammasomes. This leads to release, in a caspase-1-dependent manner, of DAMPs (including more eATP, HGMB1, and S100A9, which are potent activators of the ComC) from the cells [66–68]. We found that mice that are Nlrp3 inflammasome deficient (Nlrp3^–/–^) and caspase-1 deficient (caspase-1^–/–^) are poor mobilizers [23, 91] and, at the molecular level, these mice do not activate the ComC normally in response to pro-mobilizing agents. As already mentioned, ComC activation and release of C5a in BM blood sinusoids is important for the egress of granulocytes, which pave the way for HSPCs to egress through the BM–PB endothelial barrier [27, 56].
*-Homing and engraftment of HSPCs*. HSPCs infused into PB during transplantation are exposed to several factors released from recipient BM after myeloablative condition for transplantation. In the PB of graft recipients conditioned for transplantation, circulating activated ComC cleavage fragments, C3a and C5a, along with other mediators, activate Nlrp3 inflammasomes in HSPCs while they navigate to the recipient BM [66–68]. Activation of this intracellular PRR is important in the migration of these cells in response to BM chemoattractants (SDF-1, S1P, and eATP), which is explained by the fact that the Nlrp3 inflammasome facilitates incorporation of BM homing receptors into MLRs [28]. This mechanism explains why HSPCs from Nlrp3^–/–^ mice show impaired homing and engraftment in normal WT recipients [28]. At the same time, activation of the Nlrp3 inflammasome and the ComC in the recipient’s BM microenvironment also plays an important role in homing and engraftment of HSPCs. Specifically, both Nlrp3^–/–^ mice and C5-deficient (C5^–/–^) mice engraft poorly with normal WT BM cells [28, 29]. Figure 2 shows that activation of the Nlrp3 and ComC inflammasomes plays an important role at several steps of hematopoietic transplantation, including (i) egress/mobilization of HSPCs from BM into PB in response to pharmacological mobilization, (ii) navigation of transplanted cells in response to BM chemoattractants, and (iii) conditioning the recipient BM microenvironment to facilitate homing and engraftment of transplanted HSPCs. Thus, innate immunity has emerged as an important mediator of BM sterile inflammation during mobilization as well as during homing and engraftment of HSPCs, and modulation of innate immune responses may lead to development of more optimal clinical protocols in hematopoietic transplantations.

Finally, innate immunity, including the ComC, is an important regulator of the adaptive immune system. It is known that innate immunity delivers necessary co-stimulatory signals via antigen-presenting cells or by interacting directly with B and T lymphocytes. Consequently, innate immunity may play a role in facilitating the engraftment of transplanted HSPCs and, in some unfortunate cases, trigger a cascade of events leading to GvHD [92].

### Circadian circulation of HSPCs in PB and lymphatics—the role of innate immunity

Circadian rhythms regulate the sleep–wake cycle and repeat every 24 h. They are driven by an intrinsic circadian clock that releases HSPCs from BM into PB following circadian rhythm changes. Therefore, the number of circulating HSPCs in PB follows a circadian rhythm pattern, with the peak occurring in the early morning hours and the nadir at night [12]. We have reported that this process is regulated by changes in activation of the ComC in the early morning hours, which becomes subsequently activated due to deep-sleep hypoxia. We reported that C5^–/–^ mice do not show changes in diurnal circulation of HSPCs [27]. Moreover, inhibition of Nlrp3 inflammasomes by the small-molecule inhibitor MCC950 impaired the diurnal release of HSPCs from BM [25]. We also reported that the trigger of ComC activation is eATP release from stressed cells during deep-sleep hypoxia, which activates Nlrp3 inflammasomes to release DAMPs, which in turn activate the ComC in a P2X7- and P2X4-dependent manner [25]. Based on these circadian changes in the number of circulating cells, HSPCs are co-regulated by innate immunity signals. This has another important implication: that innate immunity regulates the circadian rhythm in the circulation of other types of cells in addition to HSPCs, including mesenchymal stromal cells (MSCs), endothelial progenitor cells (EPCs), and very small embryonic-like stem cells (VSELs) [25, 27]. Therefore, by modulating the circadian circulation of various types of stem cells in PB, innate immunity has emerged as an important guardian of tissue/organ homeostasis.

### The expression of intracellular complement proteins (complosome) regulates the metabolism and homeostasis of T cells—is it also involved in regulating HSPC metabolism?

The ComC protein components C3 and C5 are synthesized in the liver, but in addition are also expressed intracellularly in certain types of cells [15, 43], become cleaved in the cytosol to C3a and C5a, and regulate several aspects of cell metabolism and cell homeostasis [15–18]. These cytosol-expressed mediators interact with C3aR and C5aR, which are expressed intracellularly on the surfaces of lysosomes and mitochondria. The primary driver of these responses initiated by the intracellular complosome is the cell-surface antigen CD46, which binds the autocrine-secreted C3b cleavage fragment of C3 [15]. The concept of a complosome has challenged our view of the role of the ComC in regulating cell metabolism and homeostasis.

The presence of functional intracellular complement (or complosome) has been demonstrated in T lymphocytes [15–18]. As postulated by Kemper et al., C3 protein appeared during evolution in single-cell organisms to regulate metabolic and homeostatic activity. Specifically, the ancient C3 protein had several domains that played a role in fatty acid oxidation, cholesterol metabolism, and steroid metabolism [15–18]. Later in evolution, C3 expression moved mainly to hepatocytes to produce canonical C3 secreted into PB; however, some of the cells retained a gene producing a non-canonical C3 regulating metabolism and homeostasis. As reported for T lymphocytes, C3, as a non-canonically expressed protein, can be cleaved intracellularly by cathepsin L into C3a and C3b. While C3a activates intracellular C3aR, which is expressed on lysosomes to activate mTOR, the C3b fragment is secreted and engages the ComC CD46 receptor on the cell surface to (i) activate certain metabolic enzymes, (ii) increase glucose and amino acid influx, and (iii) activate intracellular C5, which releases intracellular C5a [15–18]. C5a subsequently activates intracellular C5aR on mitochondria to release ROS, which eventually leads to activation of the Nlrp3 inflammasome [15–18]. Interestingly, the engagement of lymphocyte function-associated antigen 1 (LFA-1) on the surface of T lymphocytes by ICAM-1 expressed by endothelial cells induces high cytoplasmic expression of the C3 gene in an AP-1-dependent manner. LFA-1 is an adhesion molecule involved in lymphocyte migration [15–18]. Since LFA-1 also plays a role in trans-endothelial migration of HSPCs during homing of these cells to BM or their egress from BM into PB [93], the same phenomenon could be involved in enhancing intracellular complosome expression in these cells. This possibility, however, requires further study.

**Fig. 3:**
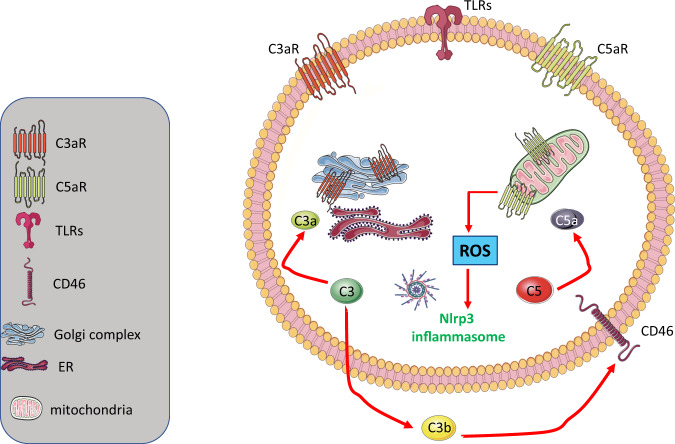
Potential expression of a functional complosome in normal HSPCs as a challenging topic for further investigations. The expression of ComC proteins and certain receptors in T lymphocytes that regulate their activity and metabolic state have been named the “complosome”. Our preliminary results indicate that the complosome may also be operational in normal murine and human HSPCs. The figure shows potential elements of the complosome that could operate in HSPCs, similarly as the elements described by others for T cells. The presence of CD46 on the surface of murine cells is still under debate.

This sequence of events mediated by intracellularly expressed ComC proteins, as described for T lymphocytes, could be relevant for hematopoiesis (Fig. 3). Our preliminary results indicate that human and murine HSPCs also express complosome elements and HSPCs from C5^–/–^ mice upon stimulation with a cocktail of hematopoietic growth factors and cytokines and reduced expression of enzymes involved in glycolysis, amino acid synthesis, and metabolism of lipids (manuscript in preparation) compared with wild type HSPCs. Since HSPCs require energy provided by glycolysis and oxidative phosphorylation during proliferation, complosomes expressed in these cells, as in T lymphocytes, may play an important role in the expansion of these cells, for example, in emergency hematopoiesis or after reconstitution of the BM microenvironment after hematopoietic transplantation [15–18]. In fact, we observed that cells from C5^–/–^ mice show impaired engraftment potential compared with wild type HSPCs in normal recipients  (manuscript in preparation).

These observations and the fact that ROS released from mitochondria in a complosome activation-dependent manner enhances the intracellular level of Nlrp3 inflammasomes supports the concept that the complosome–Nlrp3 inflammasome interaction promotes proliferation of HSPCs [94]. In accordance with this concept, a recent report suggested that glucose influx to the developing vertebrate embryo expands murine early CD41^+^ HSPCs in hematopoietic organs, and this effect depends on Nlrp3 inflammasome activation and IL-1β release [95]. As reported, the loss of Nlrp3 inflammasome components prevented the proliferation of embryonic HSPCs. Moreover, when human iPSC-derived hemogenic cells were exposed to Nlrp3 inflammasome activators, there was a significant increase in multilineage hematopoietic colony formation, which strongly suggests that the Nlrp3 inflammasome regulates expansion of early-development hematopoietic cells [95].

This raises the question of how important complosome-dependent metabolic regulation is for these effects. Our unpublished results indicate that, in fact, Nlrp3^–/–^ mice have ~20% fewer Sca-1^+^Kit^+^Lin^–^ HSPCs in BM than do WT animals [28]. In addition, our recent results indicate that C5^–/–^ HSPCs engraft more poorly after transplantation into wild-type animals and display impaired in vitro proliferation in response to a suboptimal concentration of hematopoietic stimulators (manuscript in preparation).

### Innate immunity as a driver of hematopoietic stem cell senescence

While several innate immunity effects in the hormetic zone can be beneficial for HSPCs, prolonged activation of innate immunity in the BM microenvironment may lead over time to their senescence. This process is known as “inflammaging,” which refers to chronic, low-grade, sterile inflammation that develops in hematopoietic tissues with advanced age and is characterized by enhanced myelopoiesis, due to increased numbers of myeloid-biased HSPCs and myeloid cells and the dominance of hematopoiesis over lymphopoiesis [96, 97]. This process is facilitated by the release of several pro-inflammatory cytokines from innate immunity cells that, at non-hormetic levels, negatively affect HSPCs. Overall, inflammaging is characterized by increased activity of innate immunity and a decrease in acquired immunity. It thereby enhances the basal level of inflammation and may lead to an increased risk of myelodysplasia, myeloproliferative disorders, clonal hematopoiesis of indeterminate potential (CHIP), myeloid neoplasia, and spontaneous anemia [98, 99].

Further studies are needed to see whether inhibition of sterile inflammation in BM and, in particular, inhibition of the Nlrp3 inflammasome, which is a source of DAMPs (S100A8 and S100A9), leading to myelodysplasia and BM aging, may prevent these pathologies [96–99]. Nlrp3 inflammasomes may be aberrantly activated in senescent HSPCs by mitochondrial stress and SIRT2 inactivation [7]. This raises the possibility of slowing down aging and preventing hematopoietic pathologies by employing inhibitors of PRRs (e.g., the Nlrp3 inflammasome) or innate immunity mediators (e.g., S100A8 and S100A9) or preventing development of a chronic inflammatory state in the BM microenvironment. On the other hand, as proposed, activation of the Nlrp12 inflammasome may be beneficial in maintaining the normal function of senescent HSPCs [7].

Another unwanted pathology triggered by activation of innate immunity is acute graft-versus-host disease (GvHD) [92]. DAMPs, which are released after hematopoietic transplantation and activate Nlrp3 inflammasomes, have emerged as an inducer of this complication. It has been reported that IL-1β, which is released in an Nlrp3 inflammasome-dependent manner, activates dendritic cells and T cells into pro-inflammatory T helper cells, which have been implicated in the initiation of GvHD. Inhibiting the Nlrp3 inflammasome in an animal hematopoietic transplantation model by employing its small-molecule inhibitor MCC950 ameliorated this unwanted post-transplantation complication [92].

Finally, as proposed the low oxygen tension (3% - physioxia) in BM stem cell niches as compared to ambient atmospheric tension (21%) plays an important role in maintaining a pool of long term engrafting hematopoietic stem cells (LT-HSCs) [100]. It is why collection and processing of HSCs should be performed in low oxygen tension to prevent most primitive HSCs from exhaustion at ambient oxygen tension [100, 101]. Moreover, as reported in a recent elegant paper, aged HSCs collected and processed under hypoxic conditions possess enhanced engraftment capability during competitive transplantation analysis and contained more functional HSCs as determined by limiting dilution analysis [101]. This highly relevant clinically observation could be explained by the potential hormetic effect of ROS in cytosolic signaling. Increase in ROS level in cells exposed to extra physiologic oxygen shock/stress (EPHOSS) may over-activate as we envision, Nlrp3 inflammasome, that activated beyond hormetic zone negatively affects pool of HSCs. Nevertheless, this potential link between oxygen tension, ROS, and Nlrp3 inflammasome in maintaining pool of HSPCs requires further studies.

### Conclusion and challenging questions to be addressed

As we discussed in this perspective review, innate immunity affects the development and trafficking of HSPCs. In particular, all potential beneficial effects are seen in the hormetic zone of activated innate immunity receptors and mediators. Modulation of innate immunity components, on the one hand, may facilitate mobilization, homing, and engraftment of HSPCs, and, on the other hand, inhibition of innate immunity effects outside the hormetic zone may prevent several hematopoietic pathologies related to HSPC aging. The important question is to dissect autocrine and paracrine innate immunity signals and identify all the mediators in the observed phenomena. The novel idea here is that of the complosome and the role of intracellular complement. This concept and exciting data demonstrated in T lymphocytes [15, 43] can be extended to innate immunity cells and, even more importantly, to HSPCs [8]. Our preliminary results confirm the potential involvement of intracellular C5 in regulating the metabolism of murine HSPCs. Further studies are needed to assess involvement of the complosome in the proliferation of murine and human HSPCs, and our laboratory is currently investigating these effects.

## References

[1] Dzierzak E, Bigas A (2018). Blood development: hematopoietic stem cell dependence and independence. Cell Stem Cell.

[2] Ling KW, Dzierzak E (2002). Ontogeny and genetics of the hemato/lymphopoietic system. Curr Opin Immunol.

[3] Capitano ML (2019). Toll-like receptor signaling in hematopoietic stem and progenitor cells. Curr Opin Hematol.

[4] Ratajczak MZ, Kucia M (2021). The Nlrp3 inflammasome - the evolving story of its positive and negative effects on hematopoiesis. Curr Opin Hematol.

[5] Boettcher S, Manz MG (2017). Regulation of inflammation and infection-driven hematopoiesis. Trends Immunol.

[6] Takeuchi O, Akira S (2010). Pattern recognition receptors and inflammation. Cell.

[7] Luo H, Mu WC, Karki R, Chiang HH, Mohrin M, Shin JJ (2019). Mitochondrial stress-initiated aberrant activation of the NLRP3 inflammasome regulates the functional deterioration of hematopoietic stem cell aging. Cell Rep.

[8] Ratajczak MZ, Bujko K, Cymer M, Thapa A, Adamiak M, Ratajczak J (2020). The Nlrp3 inflammasome as a “rising star” in studies of normal and malignant hematopoiesis. Leukemia.

[9] Croker BA, Silke J, Gerlic M (2015). Fight or flight: regulation of emergency hematopoiesis by pyroptosis and necroptosis. Curr Opin Hematol.

[10] Jaiswal S (2020). Clonal hematopoiesis and nonhematologic disorders. Blood.

[11] Lussana F, Rambaldi A (2017). Inflammation and myeloproliferative neoplasms. J Autoimmun.

[12] Stenzinger M, Karpova D, Unterrainer C, Harenkamp S, Wiercinska E, Hoerster K (2019). Hematopoietic-extrinsic cues dictate circadian redistribution of mature and immature hematopoietic cells in blood and spleen. Cells.

[13] Karpova D, Rettig MP, DiPersio JF. Mobilized peripheral blood: an updated perspective. *F1000Res*. 2019;8:F1000. 10.12688/f1000research.21129.1 PMC6971844 32025285

[14] Pelus LM, Broxmeyer HE (2018). Peripheral blood stem cell mobilization; a look ahead. Curr Stem Cell Rep.

[15] West EE, Kunz N, Kemper C (2020). Complement and human T cell metabolism: location, location, location. Immunol Rev.

[16] Arbore G, Kemper C, Kolev M (2017). Intracellular complement - the complosome - in immune cell regulation. Mol Immunol.

[17] Rahman J, Singh P, Merle NS, Niyonzima N, Kemper C (2021). Complement’s favourite organelle-mitochondria?. Br J Pharm.

[18] Kunz N, Kemper C (2021). Complement has brains-do intracellular complement and immunometabolism cooperate in tissue homeostasis and behavior?. Front Immunol.

[19] Hajishengallis G, Reis ES, Mastellos DC, Ricklin D, Lambris JD (2017). Novel mechanisms and functions of complement. Nat Immunol.

[20] Reis ES, Mastellos DC, Hajishengallis G, Lambris JD (2019). New insights into the immune functions of complement. Nat Rev Immunol.

[21] Huber-Lang M, Lambris JD, Ward PA (2018). Innate immune responses to trauma. Nat Immunol.

[22] Vivier E, Artis D, Colonna M, Diefenbach A, Di Santo JP, Eberl G (2018). Innate lymphoid cells: 10 years on. Cell.

[23] Thapa A, Adamiak M, Bujko K, Ratajczak J, Abdel-Latif AK, Kucia M (2021). Danger-associated molecular pattern molecules take unexpectedly a central stage in Nlrp3 inflammasome-caspase-1-mediated trafficking of hematopoietic stem/progenitor cells. Leukemia.

[24] Budkowska M, Ostrycharz E, Wojtowicz A, Marcinowska Z, Woźniak J, Ratajczak MZ (2018). A circadian rhythm in both complement cascade (ComC) activation and sphingosine-1-phosphate (S1P) levels in human peripheral blood supports a role for the ComC-S1P axis in circadian changes in the number of stem cells circulating in peripheral blood. Stem Cell Rev Rep.

[25] Adamiak M, Ciechanowicz A, Skoda M, Cymer M, Tracz M, Xu B (2020). Novel evidence that purinergic signaling - Nlrp3 inflammasome axis regulates circadian rhythm of hematopoietic stem/progenitor cells circulation in peripheral blood. Stem Cell Rev Rep.

[26] Borkowska S, Suszynska M, Mierzejewska K, Ismail A, Budkowska M, Salata D (2014). Novel evidence that crosstalk between the complement, coagulation and fibrinolysis proteolytic cascades is involved in mobilization of hematopoietic stem/progenitor cells (HSPCs). Leukemia.

[27] Borkowska S, Suszynska M, Ratajczak J, Ratajczak MZ (2016). Evidence of a pivotal role for the distal part of the complement cascade in the diurnal release of hematopoietic stem cells Into peripheral blood. Cell Transpl.

[28] Adamiak M, Abdel-Latif A, Bujko K, Thapa A, Anusz K, Tracz M (2020). Nlrp3 inflammasome signaling regulates the homing and engraftment of hematopoietic stem cells (HSPCs) by enhancing incorporation of CXCR4 receptor into membrane lipid rafts. Stem Cell Rev Rep.

[29] Kim CH, Wu W, Wysoczynski M, Abdel-Latif A, Sunkara M, Morris A (2012). Conditioning for hematopoietic transplantation activates the complement cascade and induces a proteolytic environment in bone marrow: a novel role for bioactive lipids and soluble C5b-C9 as homing factors. Leukemia.

[30] Adamiak M, Cymer M, Anusz K, Tracz M, Ratajczak MZ (2020). A novel evidence that mannan binding lectin (MBL) pathway of complement cascade activation is involved in homing and engraftment of hematopoietic stem progenitor cells (HSPCs). Stem Cell Rev Rep.

[31] Wysoczynski M, Reca R, Lee H, Wu W, Ratajczak J, Ratajczak MZ (2009). Defective engraftment of C3aR^-/-^ hematopoietic stem progenitor cells shows a novel role of the C3a-C3aR axis in bone marrow homing. Leukemia.

[32] Reca R, Mastellos D, Majka M, Marquez L, Ratajczak J, Franchini S (2003). Functional receptor for C3a anaphylatoxin is expressed by normal hematopoietic stem/progenitor cells, and C3a enhances their homing-related responses to SDF-1. Blood.

[33] Ratajczak MZ, Adamiak M, Plonka M, Abdel-Latif A, Ratajczak J (2018). Mobilization of hematopoietic stem cells as a result of innate immunity-mediated sterile inflammation in the bone marrow microenvironment-the involvement of extracellular nucleotides and purinergic signaling. Leukemia.

[34] Cymer M, Brzezniakiewicz-Janus K, Bujko K, Thapa A, Ratajczak J, Anusz K (2020). Pannexin-1 channel “fuels” by releasing ATP from bone marrow cells a state of sterile inflammation required for optimal mobilization and homing of hematopoietic stem cells. Purinergic Signal.

[35] Calabrese EJ (2018). Hormesis: path and progression to significance. Int J Mol Sci.

[36] Schirrmacher V (2021). Less can be more: the hormesis theory of stress adaptation in the global biosphere and its implications. Biomedicines.

[37] Woodruff TM, Nandakumar KS, Tedesco F (2011). Inhibiting the C5-C5a receptor axis. Mol Immunol.

[38] Detsika MG, Duann P, Atsaves V, Papalois A, Lianos EA (2016). Heme oxygenase 1 up-regulates glomerular decay accelerating factor expression and minimizes complement deposition and injury. Am J Pathol.

[39] Ratajczak MZ, Adamiak M, Ratajczak J, Kucia M (2021). Heme oxygenase 1 (HO-1) as an inhibitor of trafficking of normal and malignant hematopoietic stem cells - clinical and translational implications. Stem Cell Rev Rep.

[40] Detsika MG, Lianos EA (2021). Regulation of complement activation by heme oxygenase-1 (HO-1) in kidney injury. Antioxid.

[41] Kinderlerer AR, Pombo Gregoire I, Hamdulay SS, Ali F, Steinberg R, Silva G (2009). Heme oxygenase-1 expression enhances vascular endothelial resistance to complement-mediated injury through induction of decay-accelerating factor: a role for increased bilirubin and ferritin. Blood.

[42] Schröder-Braunstein J, Kirschfink M (2019). Complement deficiencies and dysregulation: pathophysiological consequences, modern analysis, and clinical management. Mol Immunol.

[43] Liszewski MK, Atkinson JP (2015). Complement regulator CD46: genetic variants and disease associations. Hum Genomics.

[44] Hayat SMG, Bianconi V, Pirro M, Jaafari MR, Hatamipour M, Sahebkar A (2020). CD47: role in the immune system and application to cancer therapy. Cell Oncol.

[45] Bujko K, Cymer M, Adamiak M, Ratajczak MZ (2019). An overview of novel unconventional mechanisms of hematopoietic development and regulators of hematopoiesis - a roadmap for future investigations. Stem Cell Rev Rep.

[46] Rossi L, Salvestrini V, Ferrari D, Di Virgilio F, Lemoli RM (2012). The sixth sense: hematopoietic stem cells detect danger through purinergic signaling. Blood.

[47] Ratajczak MZ, Lee H, Wysoczynski M, Wan W, Marlicz W, Laughlin MJ (2010). Novel insight into stem cell mobilization-plasma sphingosine-1-phosphate is a major chemoattractant that directs the egress of hematopoietic stem progenitor cells from the bone marrow and its level in peripheral blood increases during mobilization due to activation of complement cascade/membrane attack complex. Leukemia.

[48] Golan K, Vagima Y, Ludin A, Itkin T, Cohen-Gur S, Kalinkovich A (2012). S1P promotes murine progenitor cell egress and mobilization via S1P1-mediated ROS signaling and SDF-1 release. Blood.

[49] Juarez JG, Harun N, Thien M, Welschinger R, Baraz R, Pena AD (2012). Sphingosine-1-phosphate facilitates trafficking of hematopoietic stem cells and their mobilization by CXCR4 antagonists in mice. Blood.

[50] Ratajczak MZ (2015). A novel view of the adult bone marrow stem cell hierarchy and stem cell trafficking. Leukemia.

[51] Brunstein CG, McKenna DH, DeFor TE, Sumstad D, Paul P, Weisdorf DJ (2013). Complement fragment 3a priming of umbilical cord blood progenitors: safety profile. Biol Blood Marrow Transpl.

[52] Ratajczak MZ, Kim CH, Abdel-Latif A, Schneider G, Kucia M, Morris AJ (2012). A novel perspective on stem cell homing and mobilization: review on bioactive lipids as potent chemoattractants and cationic peptides as underappreciated modulators of responsiveness to SDF-1 gradients. Leukemia.

[53] Ratajczak MZ, Adamiak M (2015). Membrane lipid rafts, master regulators of hematopoietic stem cell retention in bone marrow and their trafficking. Leukemia.

[54] Wu W, Kim CH, Liu R, Kucia M, Marlicz W, Greco N (2012). The bone marrow-expressed antimicrobial cationic peptide LL-37 enhances the responsiveness of hematopoietic stem progenitor cells to an SDF-1 gradient and accelerates their engraftment after transplantation. Leukemia.

[55] Bertolotto M, Contini P, Ottonello L, Pende A, Dallegri F, Montecucco F (2014). Neutrophil migration towards C5a and CXCL8 is prevented by non-steroidal anti-inflammatory drugs via inhibition of different pathways. Br J Pharm.

[56] Lee HM, Wu W, Wysoczynski M, Liu R, Zuba-Surma EK, Kucia M (2009). Impaired mobilization of hematopoietic stem/progenitor cells in C5-deficient mice supports the pivotal involvement of innate immunity in this process and reveals novel promobilization effects of granulocytes. Leukemia.

[57] Yamashita M, Passegué E (2019). TNF-α coordinates hematopoietic stem cell survival and myeloid regeneration. Cell Stem Cell.

[58] Essers MA, Offner S, Blanco-Bose WE, Waibler Z, Kalinke U, Duchosal MA (2009). IFN alpha activates dormant haematopoietic stem cells in vivo. Nature.

[59] Smith JN, Kanwar VS, MacNamara KC (2016). Hematopoietic stem cell regulation by type I and II interferons in the pathogenesis of acquired aplastic anemia. Front Immunol.

[60] Yazdi AS, Ghoreschi K (2016). The interleukin-1 family. Adv Exp Med Biol.

[61] Frede S, Fandrey J, Pagel H, Hellwig T, Jelkmann W (1997). Erythropoietin gene expression is suppressed after lipopolysaccharide or interleukin-1 beta injections in rats. Am J Physiol.

[62] Madu AJ, Ughasoro MD (2017). Anaemia of chronic disease: an in-depth review. Med Princ Pr.

[63] Yáñez A, Goodridge HS, Gozalbo D, Gil ML (2013). TLRs control hematopoiesis during infection. Eur J Immunol.

[64] Garred P, Genster N, Pilely K, Bayarri-Olmos R, Rosbjerg A, Ma YJ (2016). A journey through the lectin pathway of complement-MBL and beyond. Immunol Rev.

[65] Burberry A, Zeng MY, Ding L, Wicks I, Inohara N, Morrison SJ (2014). Infection mobilizes hematopoietic stem cells through cooperative NOD-like receptor and Toll-like receptor signaling. Cell Host Microbe.

[66] Kelley N, Jeltema D, Duan Y, He Y (2019). The NLRP3 inflammasome: an overview of mechanisms of activation and regulation. Int J Mol Sci.

[67] Man SM, Kanneganti TD (2015). Regulation of inflammasome activation. Immunol Rev.

[68] Lamkanfi M, Kanneganti TD (2010). Nlrp3: an immune sensor of cellular stress and infection. Int J Biochem Cell Biol.

[69] Hu B, Jin CH, Li HB, Tong J, Ouyang X, Cetinbas NM (2016). The DNA-sensing AIM2 inflammasome controls radiation-induced cell death and tissue injury. Science.

[70] Esplin BL, Shimazu T, Welner RS, Garrett KP, Nie L, Zhang Q (2011). Chronic exposure to a TLR ligand injures hematopoietic stem cells. J Immunol.

[71] Zhao Y, Ling F, Wang HC, Sun XH (2013). Chronic TLR signaling impairs the long-term repopulating potential of hematopoietic stem cells of wild type but not Id1 deficient mice. PLoS ONE.

[72] Megías J, Yáñez A, Moriano S, O’Connor JE, Gozalbo D, Gil ML (2012). Direct Toll-like receptor-mediated stimulation of hematopoietic stem and progenitor cells occurs in vivo and promotes differentiation toward macrophages. Stem Cells.

[73] Haggadone MD, Grailer JJ, Fattahi F, Zetoune FS, Ward PA (2016). Bidirectional crosstalk between C5a receptors and the NLRP3 inflammasome in macrophages and monocytes. Mediators Inflamm.

[74] Ward PA, Fattahi F (2019). New strategies for treatment of infectious sepsis. J Leukoc Biol.

[75] Kim SY, Son M, Lee SE, Park IH, Kwak MS, Han M (2018). High-mobility group box 1-induced complement activation causes sterile inflammation. Front Immunol.

[76] Minutoli L, Puzzolo D, Rinaldi M, Irrera N, Marini H, Arcoraci V (2016). ROS-mediated NLRP3 inflammasome activation in brain, heart, kidney, and testis ischemia/reperfusion injury. Oxid Med Cell Longev.

[77] Ratajczak MZ, Adamiak M, Thapa A, Bujko K, Brzezniakiewicz-Janus K, Lenkiewicz AM (2019). NLRP3 inflammasome couples purinergic signaling with activation of the complement cascade for the optimal release of cells from bone marrow. Leukemia.

[78] Baroja-Mazo A, Martín-Sánchez F, Gomez AI, Martínez CM, Amores-Iniesta J, Compan V (2014). The NLRP3 inflammasome is released as a particulate danger signal that amplifies the inflammatory response. Nat Immunol.

[79] Ratajczak MZ, Bujko K, Ciechanowicz A, Sielatycka K, Cymer M, Marlicz W (2021). SARS-CoV-2 entry receptor ACE2 is expressed on very small CD45 ^-^ precursors of hematopoietic and endothelial cells and in response to virus spike protein activates the Nlrp3 inflammasome. Stem Cell Rev Rep.

[80] Ropa J, Cooper S, Capitano ML, Van’t Hof W, Broxmeyer HE (2021). Human hematopoietic stem, progenitor, and immune cells respond ex vivo to SARS-CoV-2 spike protein. Stem Cell Rev Rep.

[81] Kucia M, Ratajczak J, Bujko K, Adamiak M, Ciechanowicz A, Chumak V (2021). An evidence that SARS-Cov-2/COVID-19 spike protein (SP) damages hematopoietic stem/progenitor cells in the mechanism of pyroptosis in Nlrp3 inflammasome-dependent manner. Leukemia.

[82] Kovacs SB, Miao EA (2017). Gasdermins: effectors of pyroptosis. Trends Cell Biol.

[83] Hong F, Chen Y, Gao H, Shi J, Lu W, Fu C (2021). NLRP1 in bone marrow microenvironment controls hematopoietic reconstitution after transplantation. Transpl Cell Ther.

[84] Snäkä T, Fasel N (2020). Behind the Scenes: Nod-like receptor X1 controls inflammation and metabolism. Front Cell Infect Microbiol.

[85] Coutermrash-Ott S, Eden K, Allen IC (2016). Beyond the inflammasome: regulatory NOD-like receptor modulation of the host immune response following virus exposure. J Gen Virol.

[86] Massberg S, Schaerli P, Knezevic-Maramica I, Köllnberger M, Tubo N, Moseman EA (2007). Immunosurveillance by hematopoietic progenitor cells trafficking through blood, lymph, and peripheral tissues. Cell.

[87] Mazo IB, Massberg S, von Andrian UH (2011). Hematopoietic stem and progenitor cell trafficking. Trends Immunol.

[88] Ratajczak MZ, Kim CH, Wojakowski W, Janowska-Wieczorek A, Kucia M, Ratajczak J (2010). Innate immunity as orchestrator of stem cell mobilization. Leukemia.

[89] Ratajczak MZ, Adamiak M, Bujko K, Thapa A, Pensato V, Kucia M (2020). Innate immunity orchestrates the mobilization and homing of hematopoietic stem/progenitor cells by engaging purinergic signaling-an update. Purinergic Signal.

[90] Adamiak M, Lenkiewicz AM, Cymer M, Kucia M, Ratajczak J, Ratajczak MZ (2019). Novel evidence that an alternative complement cascade pathway is involved in optimal mobilization of hematopoietic stem/progenitor cells in Nlrp3 inflammasome-dependent manner. Leukemia.

[91] Lenkiewicz AM, Adamiak M, Thapa A, Bujko K, Pedziwiatr D, Abdel-Latif AK (2019). The Nlrp3 inflammasome orchestrates mobilization of bone marrow-residing stem cells into peripheral blood. Stem Cell Rev Rep.

[92] Jankovic D, Ganesan J, Bscheider M, Stickel N, Weber FC, Guarda G (2013). The Nlrp3 inflammasome regulates acute graft-versus-host disease. J Exp Med.

[93] Buffone A, Anderson NR, Hammer DA (2018). Migration against the direction of flow is LFA-1-dependent in human hematopoietic stem and progenitor cells. Cell Sci.

[94] Yang L, Hu M, Lu Y, Han S, Wang J (2021). Inflammasomes and the maintenance of hematopoietic homeostasis: new perspectives and opportunities. Molecules.

[95] Frame JM, Kubaczka C, Long TL, Esain V, Soto RA, Hachimi M (2020). Metabolic regulation of inflammasome activity controls embryonic hematopoietic stem and progenitor cell production. Dev Cell.

[96] Kovtonyuk LV, Fritsch K, Feng X, Manz MG, Takizawa H (2016). Inflamm-aging of hematopoiesis, hematopoietic stem cells, and the bone marrow microenvironment. Front Immunol.

[97] Zhao J, Ghimire A, Liesveld J (2021). Marrow failure and aging: the role of “Inflammaging”. Best Pr Res Clin Haematol.

[98] Basiorka AA, McGraw KL, Eksioglu EA, Chen X, Johnson J, Zhang L (2016). The NLRP3 inflammasome functions as a driver of the myelodysplastic syndrome phenotype. Blood.

[99] Lambert C, Wu Y, Aanei C (2016). Bone marrow immunity and myelodysplasia. Front Oncol.

[100] Mantel CR, O’Leary HA, Chitteti BR, Huang X, Cooper S, Hangoc G (2015). Enhancing hematopoietic stem cell transplantation efficacy by mitigating oxygen shock. Cell.

[101] Capitano ML, Mohamad SF, Cooper S, Guo B, Huang X, Gunawan AM (2021). Mitigating oxygen stress enhances aged mouse hematopoietic stem cell numbers and function. J Clin Invest.

